# A fatal case of *Aspergillus* endocarditis in a pediatric patient with STING-associated vasculopathy

**DOI:** 10.70962/jhi.20250262

**Published:** 2026-09-22

**Authors:** Ambre Dethier, Fanny Bajolle, Paul Bastard, Laureline Berteloot, Romain Lévy, Filippo Consonni, Quentin Deletang, Eric Dannaoui, Olivier Raisky, Philippe Drabent, Jacinta Bustamante, Fréderic Rieux-Laucat, Anne Puel, Fanny Lanternier, Marie-Louise Frémond, Bénédicte Neven

**Affiliations:** 1 Université Paris Cité, Paris, France; 2 Pediatric Hematology-Immunology and Rheumatology Unit, Necker-Enfants Malades Hospital, Assistance Publique-Hôpitaux de Paris, Paris, France; 3 M3C-Paediatric Cardiology Center, Necker-Enfants Malades Hospital, Assistance Publique-Hôpitaux de Paris, Paris, France; 4 Laboratory of Human Genetics of Infectious Diseases, Necker Branch, INSERM U1163, Imagine Institute, Paris, France; 5 St. Giles Laboratory of Human Genetics of Infectious Diseases, Rockefeller Branch, The Rockefeller University, New York, NY, USA; 6 Department of Pediatric Radiology, Necker-Enfants Malades Hospital, Assistance Publique-Hôpitaux de Paris, Paris, France; 7 Unité de Parasitologie-Mycologie, Service de Microbiologie, Hôpital Européen Georges-Pompidou, Assistance Publique-Hôpitaux de Paris, Paris, France; 8 Faculté de Santé, UR Dynamic 7380, UPEC, EnvA, USC ANSES, F-94000, Créteil, France; 9 Department of Pediatric Cardiac Surgery, Necker-Enfants Malades Hospital, Assistance Publique-Hôpitaux de Paris, Paris, France; 10 Department of Pathology, Necker Hospital, Assistance Publique-Hôpitaux de Paris, Paris, France; 11 Centre D’étude des Déficits Immunitaires, Necker-Enfants Malades Hospital, Assistance Publique-Hôpitaux de Paris, Paris, France; 12 Immunogenetic of Autoimmune Diseases, INSERM UMR 1163, Institut Imagine, Paris, France; 13 Department of Infectious Diseases and Tropical Medicine, Necker-Enfants Malades Hospital, Assistance Publique-Hôpitaux de Paris, Paris, France; 14 National Reference Center for Invasive Mycoses and Antifungals, Institut Pasteur, Université Paris Cité, Paris, France; 15 Laboratory of Neurogenetics and Neuroinflammation, Imagine Institute, INSERM, UMR1163, Paris, France; 16 Centre de Référence Maladies Rares Reference Centre for Inflammatory Rheumatism, Autoimmune Diseases and Systemic Interferonopathies in Children, Paris, France; 17 Department of Clinical and Experimental Sciences, University of Brescia, Brescia, Italy; 18 Pediatric Immunology Unit, Pediatrics Clinic, ASST Spedali Civili di Brescia, Brescia, Italy

## Abstract

SAVI is a rare *STING1* gain-of-function disorder causing systemic inflammation, vasculopathy, and interstitial lung disease. JAK inhibitors are first-line therapy. We report a SAVI patient on prolonged JAK inhibitors who developed opportunistic infections, including fatal *Aspergillus* endocarditis, underscoring therapeutic challenges.

## Teaser

Dethier et al. describe a young girl with SAVI on prolonged JAK inhibitors who developed fatal *Aspergillus* endocarditis. This report illustrates the major therapeutic challenges in STING1 gain-of-function disorders due to their intrinsic immunological abnormalities and highlights the need for more targeted alternatives such as anifrolumab.

## Introduction

STING-associated vasculopathy with onset in infancy (SAVI) is a rare autoinflammatory disease caused by monoallelic gain-of-function (GOF) variants in the stimulator of IFN response cGAMP interactor 1 gene (*STING1*), encoding STING (1). SAVI is classified within the type I interferonopathies, a group of Mendelian disorders characterized by constitutive hyperactivation of the type I IFN pathway.

SAVI is characterized by systemic inflammation, skin vasculopathy, and progressive interstitial lung disease. This may lead to the formation of cysts and lung fibrosis, resulting in respiratory failure and death. Although infectious complications are generally not a major feature of SAVI, cutaneous or osteoarticular infections may occur, particularly in patients with severe skin vasculopathy. Occasional opportunistic infections have also been reported, such as rare *Pneumocystis jirovecii* pneumonia, including in patients without immunosuppressive therapies. JAK inhibitors (JAKi) are currently the main targeted treatment for SAVI, particularly ruxolitinib and baricitinib, based on their ability to block type I IFN receptor (IFNAR) downstream signaling, leading to partial clinical and biological responses. Mild infections have been reported in these treated patients (2). Anifrolumab, an IFNAR1-specific blocking monoclonal antibody, represents an appealing alternative treatment, although experience is currently limited.

We report the case of a young female with SAVI who, while taking long-term JAKi, experienced multiple concomitant opportunistic infections, including *Mycobacterium avium* pulmonary infection and invasive pulmonary aspergillosis, which was complicated by fatal *Aspergillus* endocarditis despite aggressive antifungal therapy. Given that the long-term effects of JAKi in SAVI are not well-established, this case highlights the possibility of unusual infectious complications, underscoring the need for careful monitoring of such patients and for safer, more targeted therapeutic strategies.

## Case report

**Figure 1.**
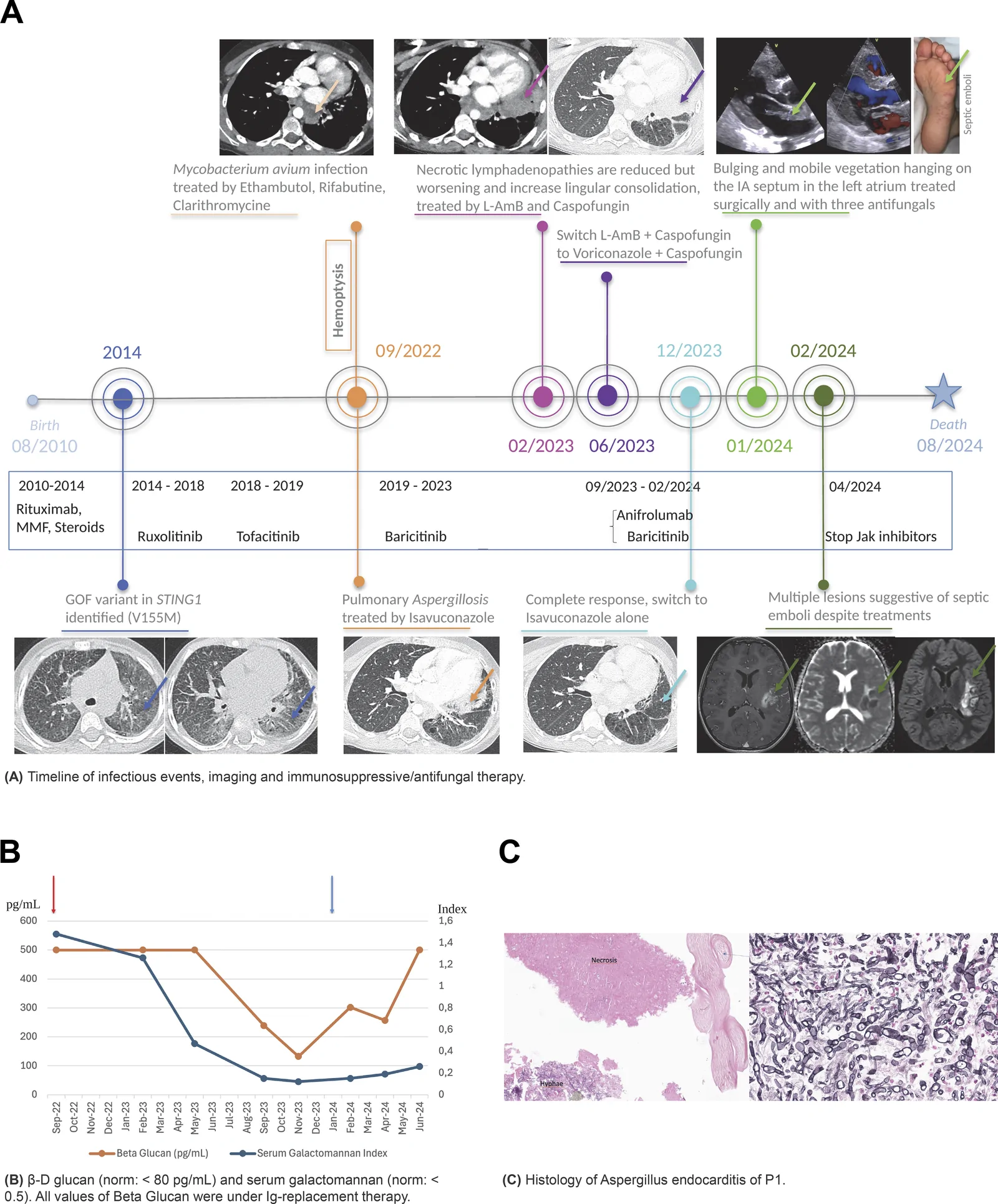
**Timeline of P1 disease course: infectious complications and successive immunosupressive and anti-infectious treatments are shown. (A)** Timeline of P1 disease course highlighting infectious complications and successive immunosuppressive and anti-infectious treatments. Diagnosis of SAVI was made in 2014 (electric blue time point); the arrow points to the interstitial lung disease with ground-glass opacities, interlobular septal thickening, intralobular lines, asymmetric distribution with left lung volume loss suggesting fibrosis, few cystic lesions, and pleural thickening. Hemoptysis occurred in July 2022 (orange time point and arrow). Chest CT in September 2022 showed left interbronchial necrotic lymphadenopathy associated with *M. avium* infection and a parenchymal consolidation of the lingula due to aspergillosis. February 2023 chest CT showed worsening and increased lingular consolidation due to aspergillosis (pink time point and arrow), while reduction of necrotic lymphadenopathy was observed. In December 2023, chest CT showed resolution of lesions (turquoise time point and arrow). January 2024 ETT demonstrated bulging, mobile vegetation attached to the interatrial septum in the left atrium (light green time point and arrow). Concomitantly, P1 foot displayed septic emboli. February 2024 brain MRI showed a left parieto-occipital intraparenchymal hematoma with perilesional oedema and multiple lesions with cocardial enhancement at the sulcal bases (dark green time point and arrows), suggestive of septic emboli. **(B)** Serum β-D-glucan and galactomannan levels over time (from September 2022 to June 2024). Of note, the patient was on IgRT, which has been reported to interfere with the β-D-glucan. Invasive *A. fumigatus* pneumonia was diagnosed in September 2022 (orange arrow). *A. fumigatus* endocarditis was diagnosed in January 2024 (blue arrow). **(C)** From left to right: H&E ×5: necrotic tissue, normal valvular tissue (arrow), and infected tissue (hyphae). Grocott ×20: septate and branching hyphae. CT, computed tomography.

The patient (P1), born to Caucasian non-consanguineous parents, was diagnosed with SAVI at the age of 4 years after identification of a germline heterozygous GOF *STING1* variant (c.463G>A/p. V155M) (1). The same variant was found in her father, paternal uncle (monozygotic twin brother of the father), and paternal grandfather, all of whom exhibited variable features of SAVI (see 1 for more details). P1 experienced systemic inflammation and progressive interstitial lung disease starting at the age of 6 mo. SAVI diagnosis led to successive treatment with ruxolitinib, tofacitinib, and then baricitinib (2). JAKi were generally well tolerated apart from one episode of JAKi withdrawal syndrome, episodes of benign respiratory viral infection, and progressive lymphopenia (Table 1). P1 was on regular Ig replacement therapy (IgRT) following rituximab therapy given in early childhood. IgRT was maintained over time due to the mild persistent hypo-IgM and the level of immunosuppression. Vaccinations were up to date, including COVID-19. She remained pauci-symptomatic until the age of 12 years, when, after 8 years of a monotherapy by JAKi, on baricitinib (dose and area under the curve [AUC] as recommended [3]), the occurrence of hemoptysis prompted further investigation. Chest computed tomography scan displayed parenchymal consolidation of the lingula and a left interbronchial necrotic lymphadenopathy (Fig. 1 A). Serum galactomannan and β-D-glucan were increased (Fig. 1 B). Wild-type *Aspergillus fumigatus* was documented by bronchoalveolar lavage and sputum (fulfilling the criteria of invasive pulmonary aspergillosis). *M. avium* was cultured from transbronchial biopsy of the necrotizing mediastinal adenopathy, confirming a co-infection. Complex anti-infectious treatment was initiated with successive antifungal drugs (as shown in Fig. 1 A), including voriconazole, isavuconazole, liposomal amphotericin B (L-AmB), and caspofungin, achieving transient microbiological and radiological control. Treatment was complicated by nephrotoxicity and subsequent emergence of azole-resistant *A. fumigatus* with new radiologic progression, requiring several therapeutic adjustments (Fig. 1 A). Therapeutic drug monitoring was performed throughout treatment. After 17 mo of curative treatment, complete resolution of parenchymal lesions and negative sputum cultures allowed secondary prophylaxis with isavuconazole. *M. avium* infection was treated with 21 mo of ethambutol, rifabutin, and clarithromycin, resulting in good radiologic outcome allowing transition to preventive treatment with azithromycin alone. Given these serious infections and the narrower spectrum of associated immunosuppression, anifrolumab was started, and the dose of baricitinib was progressively reduced to 2 mg per day (Fig. 1 A).

**Table 1.** Neutrophil and lymphocyte count over time in P1

​	Unit	Age-related reference ranges	06/2015	06/2018	06/2019	06/2020	05/2022	Age-related reference ranges	11/2022	01/2023	02/2024
Lymphocytes	/µl	1,500–6,500	3,000	1,554	**1,329**	**1,200**	1,588	1,300–6,000	**895**	**860**	**1,015**
CD3+	/µl	1,200–2,600	2,250	**979**	**877**	**852**	**1,083**	1,000–2,200	**631**	**580**	**684**
CD4+	/µl	650–1,500	1,320	**544**	**532**	**480**	**512**	530–1,300	**366**	**326**	**452**
CD8+	/µl	370–1,100	870	420	**332**	**348**	544	330–920	**255**	**242**	**209**
CD31+CD45RA+CD4+	%	43–55	*77*	*80*	*80*	*75*	*74.7*	43–55	*76.6*	*77.6*	*69*
CD45RA+CCR7+CD8+	%	52–68	*94*	65.3	67.3	67	**40.1**	52–68	**37.5**	**44**	*74.2*
CD19+	/µl	273–860	720	528	425	336	377	219–509	**127**	**184**	296
NK+	/µl	100–480	**30**	**31**	**27**	**12**	127	100–480	135	**94**	**30.45**
Neutrophils	/µl	1,800–8,000	6,300	5,700	3,800	2,300	4,400	2,000–8,000	5,900	6,940	7,060
IgG	g/L	5.82–11.54	12.19*	12.76*	15.72*	14.38*	17.98*	6.6–15.3	23.8*	21.63*	15.61*
IgA	g/L	0.46–1.57	*2.20*	*2.98*	*4.38*	*2.78*	*4.09*	0.5–2.2	*5.64*	*5.11*	*2.57*
IgM	g/L	0.54–1.55	**0.32**	**0.30**	**0.43**	**0.22**	**0.27**	0.5–1.6	**0.31**	**0.27**	**0.41**
Ruxolitinib	mg/day	​	2.5-2.5	7.5-7.5	​	​	​	​	​	​	​
Tofacitinib	mg/day	​	​	​	4-4	​	​	​	​	​	​
Baricitinib	mg/day	​	​	​	​	2-2-2	3-2-3	​	4-4	4-4	2

IgG values measured under IgRT are marked with an asterisk (*). Values above reference values are marked in italics, and values below reference values are marked in bold. The the total dose of ruxo, tofa and bari each day: ex 2.5-2.5 means 2.5 mg twice a day, 2-2-2, means 2 mg 3 times per day.

After 2 mo of secondary prophylaxis with isavuconazole, she developed painful erythematous lesions of the extremities (Fig. 1 A). Transthoracic echocardiography (ETT) revealed a large, transfixing vegetation in the left atrium with a floating extension and purulent necrotic areas, while previous ETT had been normal. Cardiovascular surgery allowed subtotal resection of the lesion, which on histopathological examination showed extensive necrosis associated with a dense inflammatory infiltrate containing abundant branching septate hyphae consistent with invasive fungal infection (Fig. 1 C). Cultures of resected tissues were positive for azole-resistant *A. fumigatus* (G448S mutation in the *CYP51A* gene) that were sensitive to L-AmB and micafungin. Septic emboli were documented in the kidney and in the brain.

Intensive antifungal treatment with voriconazole, caspofungin, and L-AmB did not prevent rapid recurrence of left atrial vegetation. The daily dose of L-AmB was increased to 7.5 mg/kg/day, and voriconazole was switched to olorofim, while anifrolumab and baricitinib were stopped. Very significant improvement of cardiac involvement was documented under caspofungin, L-AmB, and olorofim before recurrence of lesions 3 mo later. Given the non-feasibility of surgical treatment and the failure of antifungal drugs, palliative care was initiated. The patient died of respiratory failure 8 mo after the diagnosis of *Aspergillus* endocarditis. During these 2 years of intensive management of opportunistic infectious complications, the underlying disease remained clinically and radiologically stable.

Reanalysis of whole-genome sequencing, dihydrorhodamine test on circulating phagocytes, and screening for anti-cytokine autoantibodies identified no additional predisposing factor. Immune phenotyping confirmed lymphopenia, with reduced memory T cells, and impaired OKT3-induced proliferation.

## Discussion

We report a 14-year-old female with SAVI, treated with JAKi for 10 years, who developed multiple concomitant opportunistic infections including *M. avium* pulmonary infection, pulmonary aspergillosis, and eventually fatal *A. fumigatus* endocarditis.

Infectious complications are not a primary feature of type I interferonopathies. However, a recent retrospective study of the infectious burden in this group of disorders conducted at our institution highlighted that SAVI is associated with the highest risk of infection, including rare cases of opportunistic infections (such as *P. jirovecii* pneumonia, pulmonary aspergillosis, and mycobacterial infections; unpublished data). The underlying causes of this increased susceptibility are likely multifactorial: involving extrinsic factors related to immunosuppression associated with the use of JAKi and factors intrinsic to SAVI per se—including immunodeficiency, immune dysregulation, and chronic lung inflammation.

JAKi are increasingly used in SAVI and have an overall favorable safety profile; however, long-term data remain limited, and monitoring is essential, particularly given the high doses often required in these conditions (2). Infections are known side effects of these drugs, with varicella zoster virus reactivation being the most frequent. Both *M. avium* and invasive aspergillosis have been reported in patients on JAKi, although with a very low incidence (4). Inhibition of JAK1 and JAK2 affects the signaling of other cytokines, especially type II IFN, which may contribute to susceptibility to mycobacterial infections. Partial response to type II IFN may also alter macrophage activation, important for fungal clearance. Moreover, the higher the dose of JAKi, the broader is the inhibition across the JAK family. Thus, decreased signaling of JAK3 may have exacerbated the lymphopenia observed in P1, potentially contributing to the *M. avium* infectious disease.

SAVI itself may also contribute to infectious susceptibility. Lymphopenia, reduced memory T cells, and impaired lymphocyte proliferation have been reported in SAVI patients and mouse models and were present in P1. No additional cause of immunodeficiency was identified.

In addition to systemic immune dysregulation, local pulmonary factors may also contribute to infectious susceptibility. Chronic lung inflammation and architectural distortion may predispose to fungal colonization and infection in SAVI.

In our case, the severity of invasive aspergillosis leading to endocarditis is striking. *Aspergillus* endocarditis is extremely rare, representing <1% of all infectious endocarditis cases and 10–24% of fungal endocarditis. *Aspergillus* endocarditis is typically observed in patients with prosthetic cardiac valves or those who are profoundly immunosuppressed (5). The challenge of co-medication and management of drug–drug interactions in our patient may have contributed to the development of azole resistance. The prognosis of *Aspergillus* endocarditis is poor; complete surgical resection and early diagnosis are critical for improving outcomes. In P1, incomplete resection led to early recurrence of endocardial lesions and emboli, contributing to the failure of medical treatment despite optimization with a triple-therapy regimen active against the strain of *Aspergillus* isolated from cardiac tissue.

This case report highlights the need for careful monitoring of SAVI patients, and more broadly patients with other type I interferonopathies, treated with JAKi, and emphasizes the need for more targeted therapies. Anifrolumab, a monoclonal anti-IFNAR1 antibody, represents a promising alternative. A better understanding of the mechanisms underlying the intrinsic immunological abnormalities observed in SAVI patients would also help to optimize treatment and monitoring of these patients.

## Consent for publication

Written informed consent was obtained from legally authorized representatives in accordance with the Declaration of Helsinki and local regulations, with approval from the institutional review board. The study was approved by the ethics committee of INSERM (RCB 2010-A00634-35 and 2008-A01078-47).
